# Cold-induced biochemical changes in leaves of two commercial clones of *Eucalyptus*

**DOI:** 10.3389/fmolb.2025.1584132

**Published:** 2025-06-04

**Authors:** Patricia Basile, Federico Wallace, Cristina Olivaro, Nicolás De Palma, Omar Borsani, Arthur Fett-Neto

**Affiliations:** ^1^ Espacio de Biología Vegetal del Noreste, CENUR Noreste, Universidad de la República, Tacuarembó, Uruguay; ^2^ Espacio de Ciencia y Tecnología Química, CENUR Noreste, Universidad de la República, Tacuarembó, Uruguay; ^3^ Plant Physiology Laboratory, Center for Biotechnology, Federal University of Rio Grande Do Sul (UFRGS), Porto Alegre, Brazil; ^4^ Departamento de Biología Vegetal, Facultad de Agronomía, Universidad de la República, Montevideo, Uruguay

**Keywords:** *Eucalyptus grandis*, *Eucalyptus dunnii*, cold stress, biochemical responses, metabolomic

## Abstract

**Introduction:**

Cold weather poses a significant challenge to the growth of crops and subtropical tree species like *Eucalyptus*. Exposure of plants to stressful temperatures generates changes in their physiology resulting from modifications in gene expression and extensive metabolic reorganization. A direct comparison of several biochemical changes under cold exposure of leaf tissues of *E. dunnii* and *E. grandis* clones was carried out.

**Methods:**

Leaf discs of *E. grandis* and *E. dunnii* were initially maintained for 24 h at 25°C and then 4 days at 6°C to induce cold stress. Sampling was conducted at 0 h (control condition), 2 and 4 days. Several biochemical parameters were measured, and an untargeted metabolomics approach based on ultra-high performance liquid chromatography (UHPLC) coupled to linear ion trap mass spectrometry fingerprinting was carried out.

**Results:**

Results indicated distinct cold tolerance strategies in *Eucalyptus grandis* and *Eucalyptus dunnii*. *Eucalyptus dunnii* initiated protective mechanism activation after a 2-day exposure period with the accumulation of sugars and phenolic compounds, whereas *E. grandis* did so after 4 days, accumulating proline and anthocyanins. PLS-DA based on UHPLC-MS fingerprints revealed a clear species-specific effect across the metabolome. This effect was greater than the differences between cold temperatures. Additionally, this methodology allowed the putative identification of 16 phenolic marker compounds with high discriminant potential to differentiate the cold response in these two species.

## Introduction

*Eucalyptus* L’H’er. (Myrtaceae), an Australian and Indonesian native genus, is one of the economically most essential hardwood crops worldwide. Approximately 90% of the *Eucalyptus* species planted around the world are dominated by ‘the big nine’ species (*Eucalyptus camaldulensis* Dehnh., *Eucalyptus grandis* W. Mill ex Maiden, *Eucalyptus tereticornis* Sm., *Eucalyptus globulus* Labill., *Eucalyptus nitens* (H. Deane y Maiden) Maiden, *Eucalyptus urophylla* S.T. Blake, *Eucalyptus saligna* Sm., *Eucalyptus dunnii* Maiden, and *Eucalyptus pellita* F. Muell.) and their hybrids. These species are of particular interest because of their widespread cultivation (Stanturf et al., 2013). In Uruguay, *E. dunnii* and *E. grandis* account for almost 80% of the *Eucalyptus* plantations (Ministerio de Ganadería, Agricultura y Pesca 2024).

Cold is a limiting factor for crop production and subtropical tree species such as *Eucalyptus*. Low temperatures can lead to physiological and biochemical damage, such as growth inhibition, dehydration, cell membrane damage, accumulation of reactive oxygen species (ROS), and electrolyte leakage (Thomashow, 1999; Yadav, 2010; Guo et al., 2018).

Exposure of plants to stressful temperatures generates changes in their physiology that result of modifications in gene expression and extensive metabolic reorganization. Metabolism adjustment allows plants to avoid damage such as chlorophyll loss and membrane impairment resulting in solute leakage. The protective adjustments include accumulation of compatible osmolytes, such as proline and soluble sugars, photosystem and membrane composition alterations, as well as an increase in antioxidant capacity provided by enzymatic and non-enzymatic antioxidants (Janská et al., 2010; Knaupp et al., 2011; Juszczak et al., 2016; Verslues and Sharma, 2010; Fürtauer et al., 2019). Phenolic compounds such as anthocyanins, which accumulate in vacuoles, can act as non-enzymatic antioxidants, scavenging ROS (Close et al., 2004; Pennycooke et al., 2005; Król et al., 2015; Gould et al., 2018; Oberschelp et al., 2020).

Some species such as *Eucalyptus dunnii*, *Eucalyptus benthamii* Maiden & Cambage, *Eucalyptus gunnii* Miq. and *Eucalyptus nitens* are generally considered cold tolerant. Other species such as *Eucalyptus saligna* Sm., *Eucalyptus grandis* W. Mill ex Maiden and *Eucalyptus urophylla* S.T. Blake are often regarded as cold sensitive (Jacobs, 1981; Jovanovic, Arnold, and Booth, 2000; Davidson, Battaglia, and Close, 2004; Floriani et al., 2013; Arnold et al., 2015). Selection and breeding for cold tolerance has been a major goal in eucalypt forestry for decades (Arnold et al., 2004), resulting in improved genetic materials both within species and/or by hybridization.

Previous studies have examined cold responses in *E. dunnii* and *E. grandis*. Domingues et al. (2019) showed that improved control of energy availability and sink relationships were associated with growth maintenance in young trees of *E. globulus* versus their *E. grandis* counterparts when exposed to 10°C for 24 h. Mokochinski et al. (2018) compared *E. dunnii*, *E. grandis*, and *E. pellita* saplings exposed to different temperatures (10°C, 20°C, and 30°C) for 1 week. These authors carried out growth and untargeted metabolomics analyses, showing the relevance of the latter to understand changes upon cold exposure. Oberschelp et al. (2020) examined acclimated and non-acclimated saplings of *E. dunnii*, *E. benthamii*, and *E. grandis* exposed to simulated frosts over a period of 9 h, followed by 15 days in nursery conditions to determine damage indexes. In these authors’ studies, biochemical quantifications, metabolomic analyses, and proteomic comparisons were conducted, indicating the importance of osmoprotectants and antioxidants in acclimation.

Nonetheless, the basis for cold tolerance differences between commercial clones of *E. dunnii* and *E. grandis* remains an open topic. A recent study did not find differences in cold tolerance between these species (Oberschelp et al., 2020). In addition, there is little information on cold experiments carried out under winter temperatures that are frequent in Uruguayan humid subtropical climate (Cfa, Koppen climate classification), i.e., between 0°C and 10°C (Kottek et al., 2006).

To help filling this information gap, herein a direct comparison of several biochemical changes under cold exposure of leaf tissues of *E. dunnii* and *E. grandis* clones was carried out. A leaf disk-based experimental system was proposed affording faster execution and demanding simpler resources for comparative evaluations. The main question addressed was whether these species differ in mechanisms of defense against low temperatures typically present in winter of mild temperate climates.

## Materials and methods

### Plant materials and cold treatments

Seedlings of commercial genotypes of *Eucalyptus grandis* Hill ex Maiden and *Eucalyptus dunnii* Maiden kindly donated by Lumin S.A and UPM Biofore, respectively, were used in this study.

The experiment was conducted in a growth chamber (Thermo Fisher model precision 818) under irradiance of 515 μmol s^−1^ (cold fluorescent lamps) and a photoperiod of 12 h/12 h (day/night) at the Tacuarembó campus of the Universidad de la República (UdelaR), Uruguay. For the experiment, leaf discs of plantlets were prepared as described in De Palma et al. (2023).

Briefly, healthy, fully expanded leaves were removed at the petiole insertion point, immersed in 1.5% (v/v) sodium hypochlorite for 15 min, and washed three times with distilled water. Subsequently, 1 cm diameter discs were cut using a steel cork borer and placed in 9 mm Petri dishes (30 discs per dish) with filter paper discs soaked in 0.1 × MS culture medium salts (20 mL per dish) (Murashige and Skoog, 1962).

Leaf discs were initially maintained for 24 h at 25°C and then 4 days at 6°C to induce cold stress. Sampling was conducted at 0 h (control condition, immediately before treatment application), 2 and 4 days. After harvest, disks were immediately frozen at −80°C and then liophilized. Henceforth, the term dry leaf disks refers to the ground lyophilized tissues.

### Pigment analysis

Chlorophyll A (ChlA), chlorophyll B (ChlB), carotenoids, and anthocyanins were analyzed as described in De Palma et al. (2023). Dry leaf disks (50 mg) were placed in tubes with 1 mL of cold acetone/0.1 M tris buffer (80/20, v/v) (pH 7.8). These extracts were ground and ultrasonicated in ice water for 15 min and then placed in the dark at 4°C for 24 h. The next day, they were ultrasonicated again for 15 min. The extracts were centrifuged for 15 min at 10,000 × g and 4°C, and the supernatant was recovered, diluted with 100 µL of the extraction solution, and maintained in darkness. Absorbance readings were performed in a SpectraMax M2 UV–Vis spectrophotometer (Molecular Devices, San Jose, United States) at 470, 537, 647, and 663 nm. Concentrations of ChlA, ChlB, Total chlorophyll (TChl), carotenoid, and anthocyanin were estimated according to the equations proposed by Sims and Gamon (2002). ChlA/ChlB ratios were also determined.

### Proline quantification

Proline concentration was determined according to Lee et al. (2018), with minor modifications. Dry leaf disks (50 mg) were placed in tubes with 800 µL of 3% (w/v) sulfosalicylic acid and vortexed. The extracts were centrifuged for 15 min at 16,000 × g. One hundred µL of supernatant was mixed with 200 µL of 1.25% (w/v) ninhydrin in 80% (v/v) glacial acetic acid, vortexed, and incubated for 1 h in an oven at 100°C. The reaction was stopped by transferring the mixture to ice for 10 min and vortexing. Absorbance was read in a spectrophotometer at 520 nm. Proline concentration was calculated from a standard curve and expressed on a DW basis.

### Lipid peroxidation analysis

Lipid peroxidation was analyzed according to Velikova et al. (2000) with minor modifications. One hundred mg of dry leaf disks were ground with 1,334 µL 0.1% (v/v) of trichloroacetic acid (TCA) and centrifuged at 16,000 × g at 4°C for 20 min. Then, 200 µL of supernatant were collected and mixed with 400 µL of 0.5% (w/v) thiobarbituric acid (TBA) in 20% (v/v) TCA solution. Next, the tubes were incubated in a hot water bath at 100°C for 20 min, after which the reaction was stopped in an ice bath. Finally, absorbance readings were performed at 532 and 600 nm. The lipid peroxidation was estimated as malondialdehyde (MDA) concentration using an extinction coefficient of 155 mM^−1^ cm^−1^ and expressed on a DW basis.

### Hydrogen peroxide (H_2_O_2_) analysis

The hydrogen peroxide concentration was determined from the extract used for lipid peroxidation analysis as described in Velikova et al. (2000). Twenty µL of TCA extract were mixed with 100 µL of 10 mM potassium phosphate buffer and 400 µL 1 M potassium iodide (KI). Next, the reaction was incubated at 4°C for 1 h in the dark. The absorbance was read at 390 nm in a spectrophotometer. The hydrogen peroxide concentration was calculated and expressed on a DW basis.

### Quantification of total soluble proteins (TSP)

Quantification of TSP followed the method of Bradford (1976). First, 50 mg of dry leaf disks were ground using a tissue disruptor with 1.5 mL of extraction buffer containing 1% (w/v) polyvinylpyrrolidone, 1 mM ethylenediaminetetraacetic acid, 1 mM phenylmethylsulfonyl fluoride, and 50 mM HEPES buffer (pH 7.4). Next, the ground material was centrifuged at 16,000 × g at 4°C for 15 min, and the resulting supernatants (protein extracts) were collected and transferred to new tubes. To quantify TSP, 30 μL of protein extract was mixed with 1.5 mL of Bradford reagent (PierceTM Bradford Protein Assay Kit, Thermo Scientific, Waltham, United States). Finally, absorbance readings were taken at 595 nm, and the TSP content was determined using a BSA standard curve.

### Superoxide dismutase (SOD) activity

SOD activity was determined as outlined by Beyer and Irwin (1987), with slight modifications. Thirteen µL of protein extract were combined with 1 mL of 50 mM phosphate buffer (pH 7.8) containing 57 µM nitro blue tetrazolium (NBT), 9.9 mM L-methionine, 0.025% (w/v) Triton® X-100, and 2 mM riboflavin. The mixture was then placed for 15 min in a transilluminator with fluorescent light (20 W). Absorbance readings were obtained at 560 nm, and the SOD activity was calculated and expressed as SOD units. µg^−1^ protein.

### Relative electrolyte leakage (EL%)

Relative electrolyte leakage was estimated according to Thalhammer et al. (2014), with some modifications. Previously washed leaf disks were placed in plastic tubes with ultrapure water and incubated in agitation for 24 h at room temperature. Then, the initial electric conductivity (Ci) was measured with a portable conductivity meter (HORIBA, model LaquaTwin, Kyoto, Japan). Afterwards, the tubes were incubated in a hot water bath for 30 min at 100°C. Subsequently, final electric conductivity (Cf) was measured, and EL% was calculated following the equation

$$EL\%=\left(\frac{Ci}{Cf\;}\right)\times 100$$

### Total soluble sugar content (TSS)

TSS quantification followed the protocol of Chow and Landhäusser (2004) with minor modifications. Thirteen mg of leaf disks were ground in liquid nitrogen and extracted two times in 750 µL ethanol 80% (w/v). Then, the extracts were incubated in a dry bath for 20 min at 75°C and centrifuged for 10 min at 16,000 × g. Next, the supernatants were collected and combined, constituting the total soluble sugar extracts. Afterwards, 75 µL of total soluble sugar extracts were diluted with 125 µL ethanol 80% (p/v) and mixed with 200 µL 5% (w/v) phenolic acid and 1 mL sulfuric acid. The reaction was incubated with agitation for 20 min at room temperature, and then the absorbances were read at 490 nm. The TSS concentration was expressed on a DW basis.

### Total phenolics content

Total phenolics were determined as reported by Makkar (2000). Two hundred mg of dry leaf disks were ground with 10 mL 70% acetone (v/v) and ultrasonicated in ice water for 20 min. Next, extracts were centrifuged at 3,000 × g for 10 min at 4°C. Supernatants were collected and mixed with 450 µL distilled water, 250 µL 1 N Folin-Ciocalteu reagent and 1.25 mL 20% (w/v) sodium carbonate. The reaction mixture was vortexed and incubated for 40 min in darkness. Then, absorbance readings were done at 725 nm. Total phenolics concentration was expressed as mg gallic acid g^−1^DW.

### Statistical analysis

Experiments were carried out in a totally randomized design using four biological replicates of each treatment, and statistical analysis was performed using R studio software version 2024.4.2. Normality tests and one-way Analysis of Variance (ANOVA) followed by Tukey’s, Mann–Whitney or Kruskal–Wallis tests were applied when appropriate. Multivariate analysis was performed with Principal Components Analysis (PCA) with “FactoMineR” package in R (R Core Team, 2023).

### LC-MS/MS fingerprinting with a focus on phenolic compounds

Leaf disks were extracted according to Noleto-Dias et al. (2023) with some modifications. Succinctly, 30 mg of dry leaf disks were extracted with 750 µL water: methanol (50:50, v/v) and then centrifuged at 13,000 × g for 5 min. Supernatants were collected, filtered, and placed in glass vials for analysis.

Liquid chromatography coupled with tandem mass spectrometry (LC-MS/MS) was performed using a linear ion trap mass spectrometer (LTQ XL, Thermo Scientific, San José, United States) equipped with an electrospray ionization (ESI) interface. The liquid chromatography was conducted on a UHPLC system (Ultimate 3000 RSLC, Dionex, Waltham, United States). All data were collected and processed using XcaliburTM software (v3.063) (Thermo Scientific, 2013). The parameters of the source ESI were as follows: spray voltage 3.5 kV, capillary voltage −3 V, tube lens offset −228 V, sheath gas (nitrogen) flow rate 20 (arbitrary units, a.u.), auxiliary gas (nitrogen) flow rate 20 a.u., and capillary temperature 300°C. Mass spectrometry analysis was conducted in the negative ionization mode and scanned from *m/z* 150 to 900.

The separation column was a Hypersil Gold RP C18 (100 mm × 2.1 mm ID, 1.9 µm particle size) from Thermo Scientific (Waltham, United States). A 30 µL sample aliquot was injected into the UHPLC-MS system. The mobile phase consisted of a gradient of water and acetonitrile, both with 0.1% (v/v) formic acid. Initial conditions were 5% acetonitrile, and the gradient was run to 95% acetonitrile within 25 min and returned to 5% acetonitrile in the next 12 min. The flow rate was set to 0.25 mL/min, and the column was maintained at 40°C. In LC-MS/MS, the mass spectrometry was operated under Data Dependent™ scan mode. This acquisition mode included a single Full MS scan followed by MS/MS scans on the most abundant ions. The collision energy used was 35%.

Leaf disk samples were randomly analyzed using the pre-defined UHPLC-MS system. Eight records were obtained for each sample, including biological replicates. In addition, five quality control (QC) samples, prepared with 30 µL of each analyzed sample extract, were used to evaluate the chemometric results. QC samples and extracting solvent blanks were periodically injected into the sample sequence.

The data matrices for the chemometric analysis were obtained using MZMine 3.9.0 Software (Schmid et al., 2023). Partial Least Square Discriminant Analysis (PLS-DA) was performed using the SOLO 9.3 chemometrics Software (Eigenvector Research, Manson, WA, United States). Moreover, the average of the variable importance in projection (VIP) values obtained after building PLS-DA models were used to select variables with the most significant influence on the observed sample distribution using 1.0 as the cut-off value. Based on these variables, the MS^2^ spectrum of each one was used for metabolite identification. This was achieved by searching the NIST 20 Tandem Library (lr_msms_nist and nist_msms) using the MS/MS Identity Search function of the NIST MS Search Program (v.2.4) and by comparing the results with reported data in the literature for *Eucalyptus* samples. Finally, using those metabolites previously identified, a Heatmap was generated using “pheatmap” package in R (R Core Team, 2023). Hierarchical clustering was performed using Pearson correlation.

## Results

### Time course changes in biochemical responses to cold stress in *Eucalyptus grandis* and *Eucalyptus dunnii*

**FIGURE 1:**
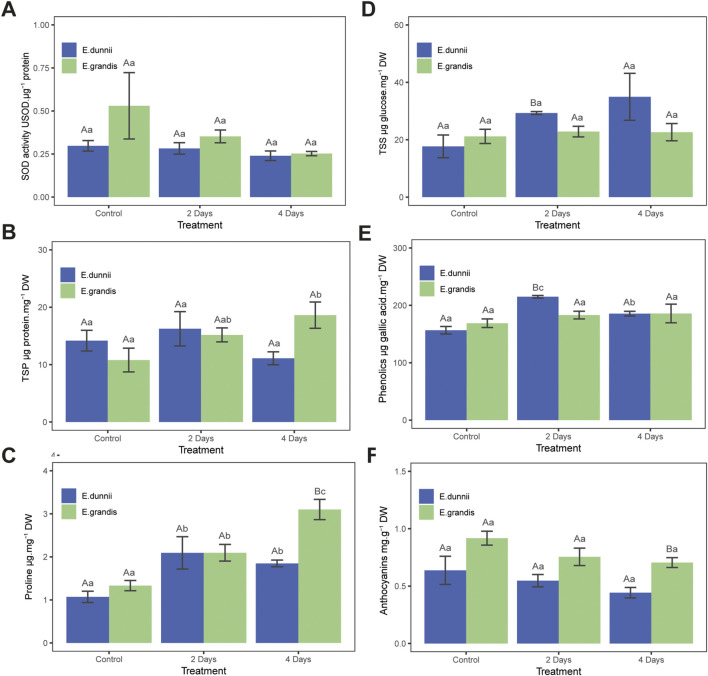
Stress protection parameters in *Eucalyptus grandis* and *Eucalyptus dunnii* leaf disks on the 2nd and 4th day of cold stress. Values correspond to mean ± SE (*n* = 4). Statistically different samples (*p* < 0.05) are indicated in capital or lowercase letters after inter-species or inter-treatment comparison, respectively. Lines on top of bars are standard errors. **(A)** Superoxide dismutase (SOD) activity. **(B)** Total soluble protein (TSP) concentration. **(C)** Proline concentration. **(D)** Total soluble sugars (TSS) concentration. **(E)** Total phenolic compounds concentration. **(F)** Anthocyanin concentration.

**FIGURE 2:**
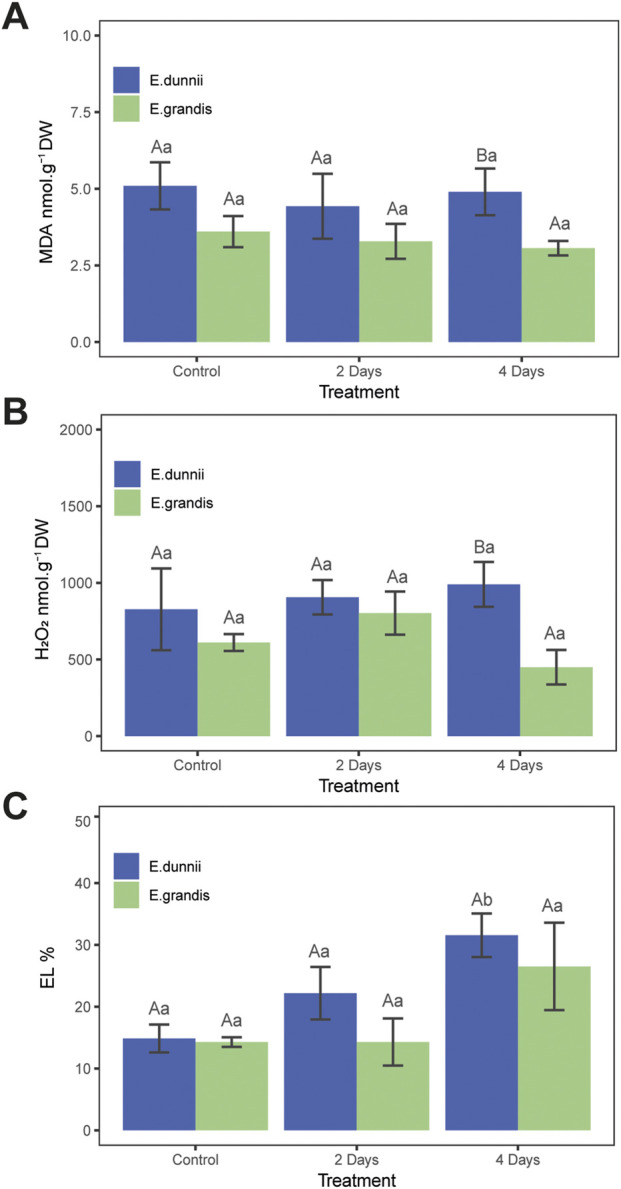
Stress indicator parameters in *Eucalyptus grandis* and *Eucalyptus dunnii* leaf disks on the 2nd and 4th day of cold stress. Values correspond to mean ± SE (*n* = 4). Statistically different samples (*p* < 0.05) are indicated in capital or lowercase letters after inter-species or inter-treatment comparison, respectively. Lines on top of bars are standard errors. **(A)** MDA levels. **(B)** H_2_O_2_ concentration. **(C)** Relative electrolyte leakage (EL%).

In *E. grandis*, significant differences were observed only in total soluble protein (TSP) and proline content across the experimental days (Figures 1B,C). TSP increased over cold exposure time, nearly doubling on day 4 (Figure 1B). From day one to the fourth, proline concentration increased significantly, approximately three-fold (Figure 1C). Cold treatment did not induce changes in photosynthetic pigments such as TChl, ChlA, ChlB, ChlA/ChlB, carotenoids, TSS, phenolics, SOD activity, anthocyanins, MDA, H_2_O_2_, or EL% (Table 1; Figures 1A,D–F, 2).

**TABLE 1:** Photosynthetic pigments in *Eucalyptus grandis* and *Eucalyptus dunnii* leaf disks on the 2nd and 4th day of cold stress.

Photosynthetic pigment		*E. grandis*		*E. dunnii*	
	Treatment	Mean ± SE	Stat	Mean ± SE	Stat
*Chlorophyll a (ChlA)*	Control	3.42 ± 0.33	Aa	2.72 ± 0.18	Aa
	Day 2	3.6 ± 0.49	Ba	2.26 ± 0.25	Aa
	Day 4	4.15 ± 0.52	Aa	2.54 ± 0.18	Aa
*Chlorophyll b (ChlB)*	Control	1.94 ± 0.12	Aa	1.62 ± 0.18	Aa
	Day 2	1.92 ± 0.19	Aa	1.35 ± 0.14	Aa
	Day 4	2.17 ± 0.26	Ba	1.42 ± 0.11	Aa
*Total Chlorophyll (TChl)*	Control	5.35 ± 0.43	Aa	4.34 ± 0.36	Aa
	Day 2	5.53 ± 0.68	Ba	3.60 ± 0.39	Aa
	Day 4	6.32 ± 0.77	Ba	3.96 ± 0.29	Aa
*Carotenoids*	Control	1.34 ± 0.08	Aa	1.24 ± 0.07	Aa
	Day 2	1.45 ± 0.14	Aa	1.15 ± 0.80	Aa
	Day 4	1.56 ± 0.17	Aa	1.16 ± 0.76	Aa
*ChlA/ChlB*	Control	1.76 ± 0.08	Aa	1.71 ± 0.06	Aa
	Day 2	1.86 ± 0.76	Aa	1.67 ± 0.04	Aa
	Day 4	1.91 ± 0.05	Aa	1.80 ± 0.05	Aa

Values determined for total chlorophyll, chlorophyll *a* and *b*, and carotenoids are expressed in milligrams per Gram of dry weight (mg.g^−1^DW). Values correspond to mean ± SE (*n* = 4). Statistically different samples (*p* < 0.05) are indicated in capital or lowercase letters after inter-species or inter-treatment comparison, respectively.

In *E. dunnii*, significant differences were observed only for EL%, proline, and phenolic compounds across the experimental days. After 4 days of stress exposure, EL values doubled, while lipid peroxidation (MDA) and H_2_O_2_ content remained relatively stable (Figure 2). Concerning protective mechanisms, the highest accumulation of the osmolyte proline was observed on day two, in which it approximately doubled its initial value, remaining stable on day 4 (Figure 1C). Total phenolic amount increased significantly over time, peaking on day two but slightly decreasing on day four (Figure 1E). Photosynthetic pigments (TChl, ChlA, ChlB, ChlA/ChlB, and carotenoids), anthocyanins, and SOD activity did not exhibit significant changes during the experimental period in this species (Table 1; Figures 1A,F).

### Comparison of biochemical changes under cold stress between species: *Eucalyptus grandis* versus *Eucalyptus dunnii*

Regarding stress indicators, there were no significant differences between species in EL% during the experiment (Figure 2C). Moreover, lipid peroxidation (MDA concentration) and H_2_O_2_ only exhibited significant differences on day 4, when *E. dunnii* showed higher values for both parameters (Figures 2A,B).

As for TChl, *E. grandis* showed higher values on day 2. Higher concentrations of ChlA and ChlB were also recorded in *E. grandis* on days 2 and 4, respectively. The ChlA/ChB ratio did not show significant differences between species (Table 1).

SOD activity, carotenoids, and TSP did not present differences between species throughout the experiments (Table 1; Figures 1A,B). After 2 days of stress, *E. dunnii* showed higher levels of TSS and phenolics (Figures 1D,E). The remaining parameters, proline and anthocyanin concentrations, displayed significant differences between species only on day 4 when *E. grandis* showed higher values than *E. dunnii* (Figures 1C,F).

### Principal component analysis points to species and treatment trends

**FIGURE 3:**
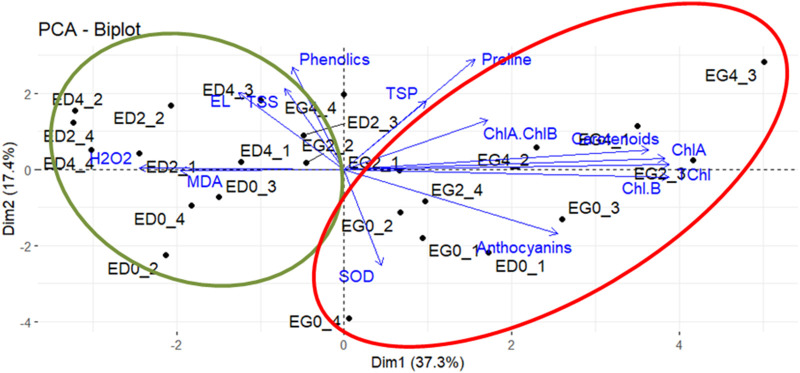
Principal components analysis. Biplot diagram for components 1 and 2 show sample observations and biochemical parameters. Vector lengths (original variables) indicates the strength of the relationship, and the angle between two vectors shows the degree of correlation. Ellipses were drawn to highlight sample observations (green ellipse: *Eucalyptus dunnii*; red ellipse: *Eucalyptus grandis*). *Eucalyptus grandis* control (EG0_1-EG0_4), *Eucalyptus grandis* on the second day of stress (EG2_1-EG2_4), *Eucalyptus grandis* on the fourth day of stress (EG4_1-EG4_4), *Eucalyptus dunnii* control (ED0_1-ED0_4), *Eucalyptus dunnii* on the second day of stress (ED2_1-ED2_4), *Eucalyptus dunnii* on the fourth day of stress (ED4_1-ED4_4).

Principal component analysis (PCA) was performed with biochemical parameter data to study the relationships between species and treatments with metabolism and stress tolerance. 2 PC (54.7% variation) were used to generate a biplot (Figure 3). PC1 explained the highest proportion of variance (37.3%) and represented differences between species. Variables like proline, TSP, anthocyanins, carotenoids, SOD activity and chlorophylls (TChl, ChlA, ChlB, ChlA/ChlB) were positively associated with PC1 and *E. grandis*. Moreover, variables like TSS, phenolics, MDA, EL% and H_2_O_2_ were negatively correlated with PC1 and associated with *E. dunnii*.

PC2 explained 17.4% of the variance and pinpointed cold treatments. EL%, TSS phenolics, TSP, proline, carotenoids and ChlA/ChlB were positively correlated with PC2 and pointed to day 2 and day 4. PC2 was negatively correlated with variables such as anthocyanins and SOD activity, associated with non-stressed condition (i.e., day zero) (Figure 3).

### Metabolomic analysis of phenolics compounds

**FIGURE 4:**
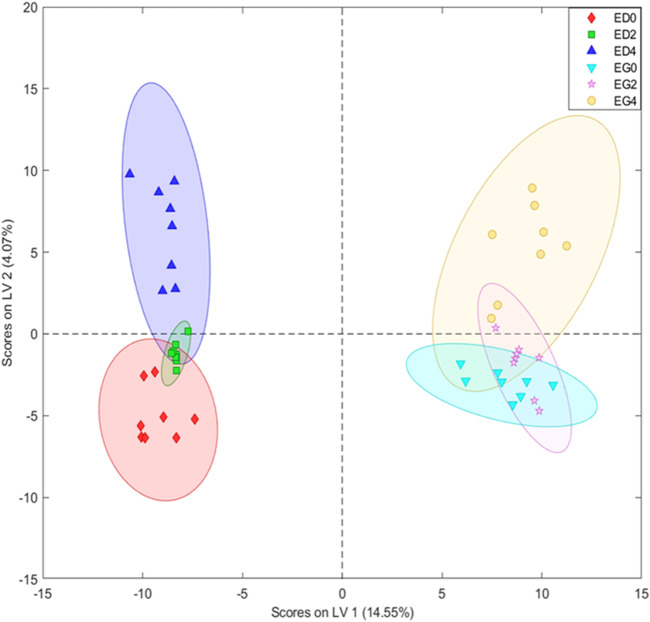
Partial least squares-discriminant analysis (PLS-DA) with eight latent variables (LVs) scores of the UHPLC-MS datasets derived from leaf disks extracts of *Eucalyptus dunnii* and *Eucalyptus grandis* maintained for 24 h at 25°C to simulate normal growth conditions and 4 days at 6°C to induce cold stress. Sampling was conducted at 0 h (control treatment), 2 and 4 days. *Eucalyptus grandis* control (EG0), *Eucalyptus grandis* on the 2nd day of stress (EG2), *Eucalyptus grandis* on the 4th day of stress (EG4), *Eucalyptus dunnii* control (ED0), *Eucalyptus dunnii* on the 2nd day of stress (ED2), *Eucalyptus dunnii* on the 4th day of stress (ED4).

A metabolomic analysis focused on obtaining a phenolic-focused fingerprint was performed to further disentangle the relationship between *Eucalyptus* species and cold treatments. The PLS-DA (8 LVs) score plot based on UHPLC-MS fingerprints revealed a clear separation between *E. grandis* and *E. dunnii* samples, as well as a distinction among stress days (days 0 and 2 vs. day 4) (Figure 4). These findings are further supported by the figure of merit in Supplementary Table S1, which shows 100% sensitivity and specificity, along with a classification accuracy of 100% within each sample group.

Additionally, the UHPLC-ESI-IT-MS^2^ experiments in data-dependent mode allowed the putative identification of 16 significant phenolic compounds with high discriminant potential to differentiate the cold response in these two species. In total, seven flavonols, four ellagitannins, two gallotanins, one flavone, one phenolic acid and one cyclitol phenolic were identified (Supplementary Figure S1). These metabolite differences constitute potential markers of cold response differences.

Seven distinct flavonols were detected in peaks L5, L7, L9, L12, L13, L14, and L15. Notably, these included myricetin glucuronide (L5), myricetin hexoside (L7), quercetin galloyl hexoside (L9), quercetin glucuronide (L12), kaempferol glucuronide (L13), kaempferol (L14), and quercetin deoxyhexoside (L15) (Table 2). Four ellagitannins were identified in peaks L1, L4, L6, and L8. These were characterized as HHDP-galloyl-hexoside (L1), ellagic acid hexoside (L4), ellagic acid pentoside (L6), and HHDP digalloyl hexoside (L8). Two gallotannins were detected in peaks L3 and L11, corresponding to trigalloylhexoside and tetragalloylhexoside, respectively. Finally, a single cyclitol phenolic, phenolic acid and flavone were identified in peaks L2, L10, and L16, respectively. Tentative identifications suggest coumaroylquinic acid as the cyclitol phenolic, ellagic acid as the phenolic acid, and apigenin glucuronide as the flavone.

**TABLE 2:** Peak assignments in *Eucalyptus dunnii* and *Eucalyptus grandis* leaf extracts by LC-ESI-IT-MS2 in negative ionization mode.

Peak number	RT (min)	[M-H]^−^ (*m/z*)	Main MS/MS fragments (*m/z*)	Identification	Compound group	VIP score
L1	2.28	633	614, 481, 421, 301, 275, 249, 231, 203	HHDP galloyl-hexoside	Ellagitanin	1.131
L2	4.45	337	191, 173, 163, 155, 119	Coumaroylquinic acid	Cyclitol phenolic	1.193
L3	5.24	635	617, 483, 465, 447, 423, 313, 295	Trigalloylhexoside	Gallotanin	1.039
L4	5.26	463	447, 419, 301	Ellagic acid hexoside	Ellagitanin	1.036
L5	5.90	493	475, 449, 389, 359, 331, 317, 299, 193, 179	Myricetin glucuronide	Flavonol	1.093
L6	6.02	433	373, 343, 301, 161	Ellagic acid pentoside	Ellagitanin	1.129
L7	6.04	479	317, 299, 287, 179, 151	Myricetin hexoside	Flavonol	1.11
L8	6.08	785	633, 615, 483, 419, 301, 275	HHDP digalloylhexoside	Ellagitanin	1.277
L9	6.16	615	489, 463, 343, 313, 301, 271, 241, 179	Quercetin galloyl hexoside	Flavonol	1.014
L10	6.26	301	283, 257, 229, 185	Ellagic acid	Phenolic acid	1.076
L11	6.37	787	635, 617, 573, 465, 449, 403, 301	Tetragalloyl hexoside	Gallotanin	1.135
L12	6.75	477	433, 301, 175, 151	Quercetin glucuronide	Flavonol	1.217
L13	7.45	461	443, 417, 327, 285, 257, 197, 175, 157	Kaempferol glucuronide	Flavonol	1.041
L14	7.47	285	257, 243, 213, 169, 151	Kaempferol	Flavonol	1.282
L15	7.52	447	429, 357, 343, 327, 321, 315, 301, 255, 179, 151	Quercetin Deoxyhexoside	Flavonol	1.118
L16	7.92	445	427, 401, 341, 311, 269, 175	Apigenin glucuronide	Flavone	1.171

Metabolites were selected based on the VIP scores (threshold of 1).

**FIGURE 5:**
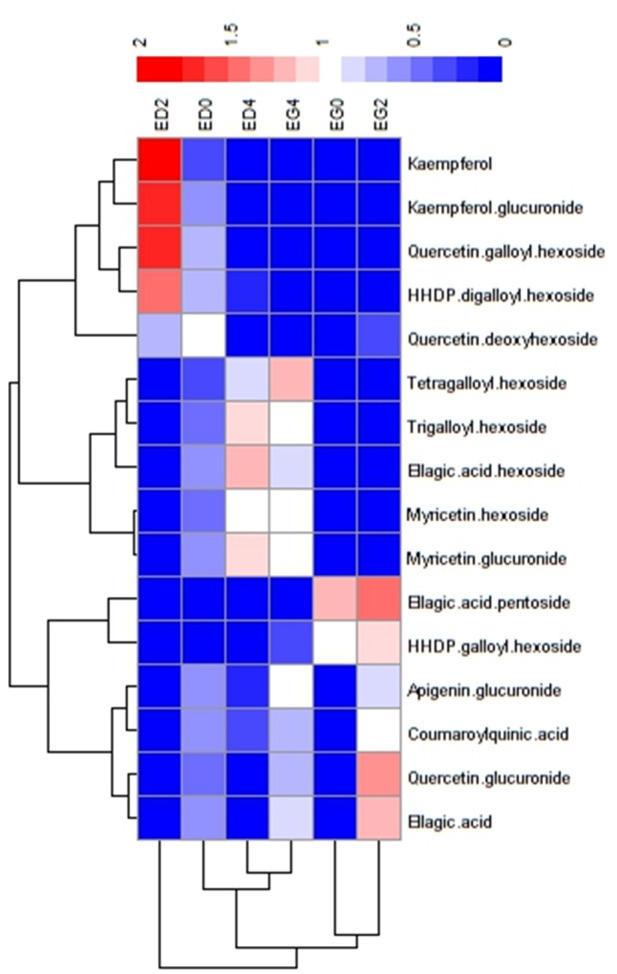
Heatmap representation of the putative identified metabolites significantly discriminant between species and treatments. Data was clustered by samples and metabolites.

To further explore the metabolic differences, heatmap and hierarchical clustering analyses were done for the 16 tentatively identified metabolites. These analyses revealed distinct patterns associated with species and cold treatment (Figure 5). Two clusters emerged from the hierarchical clustering. The first cluster grouped all *E. grandis* treatments (EG0, EG2, and EG4), while the second cluster contained only the *E. dunnii* 2-day cold treatment (ED2) (Figure 5, bottom dendrogram).

Metabolites were categorized into three groups based on abundance patterns. Group I comprised metabolites that were significantly more abundant in the *E. dunnii* control (ED0) and 2-day cold treatment (ED2) like kaempferol, kaempferol glucuronide, quercetin galloyl hexoside, quercetin deoxyhexoside and HHDP digalloylhexoside (Figure 5, lateral dendrogram). Group II included metabolites more abundant in the 4-day cold treatment of both species (ED4 and EG4) such as myricetin glucuronide, myricetin hexoside, ellagic acid hexoside, trigalloyl hexoside, and tetragalloylhexoside. Finally, Group III consisted of metabolites that were more abundant in *E. grandis* control (EG0) and 2-day cold treatment (EG2), like ellagic acid, quercetin glucuronide, coumaroylquinic acid, apigenin glucuronide, HHDP galloyl hexoside and ellagic acid pentoside.

## Discussion

### EL%, MDA, H_2_O_2_, and SOD

Over the experimental period, EL% increased only in *E. dunnii* after 4 days of cold stress, while *E. grandis* remained unaffected. Similarly, timewise the lipid peroxidation end-product MDA and H_2_O_2_ amounts did not exhibit significant changes in either species. These observations contrast with those reported by Liu et al. (2014) for *E. dunnii*, in which H_2_O_2_ levels increased by 50% after 3 h at 4°C. This discrepancy may be a function of the distinct time scales of sampling used in the two studies (hours vs. days).

Overall data from this study indicated significant interspecific differences in a limited number of parameters. Whereas no significant temporal variations in MDA and H_2_O_2_ concentrations were observed between species, *E. dunnii* exhibited elevated lipid peroxidation (MDA) and H_2_O_2_ amounts on day 4. However, *E. dunnii* appeared to maintain relatively higher basal levels of these parameters in the preceding days.

Given the absence of increased lipid peroxidation or H_2_O_2_ content throughout the assay time, the elevated EL% observed in *E. dunnii* could primarily be the result of K^+^ efflux rather than membrane damage (Palta et al., 1977; Demidchik et al., 2014; De Palma et al., 2023). Nonetheless, the loss of K^+^ may affect cell turgor and disturb growth. Alternatively, the observed increase in EL% might be attributed to alterations in membrane fluidity induced by low temperatures. In this context, a reduction in membrane fluidity is known to enhance membrane permeability, facilitating the efflux of water and solutes (Xin and Browse, 2000).

The activity of SOD remained unchanged in *E. dunnii* and *E. grandis*. These data differ from the findings of Sales et al. (2013) and Oberschelp et al. (2020) which observed an increase in SOD activity under chilling and freezing temperatures. As SOD serves as a primary defense against superoxide radicals generated during photoinhibition (Mittler, 2002) by converting them to H_2_O_2_, the stable levels of the latter could be at least partly linked to the SOD activity profile.

### Photosynthetic pigments

Under low-temperature conditions, all major components of photosynthesis, including thylakoid electron transport, the carbon reduction cycle and control of stomatal conductance, can be disrupted (Allen and Ort, 2001; Butnor et al., 2019). Plants adjust the composition of their antenna complexes and pigments to avoid photooxidative damage caused by the overproduction of ROS (Ort, 2001). In the present study, there were no significant alterations in chlorophyll and carotenoid concentrations in either species throughout the experimental period. This observation suggests defense mechanisms against cold stress were effective in both species.

A common plant strategy to mitigate photodamage involves reducing photosystem size (Mokochinski et al., 2018; Schimpl et al., 2018; Oberschelp et al., 2020; Oberschelp et al., 2022). However, our study suggested that the two species examined did not appear to employ this mechanism, as *E. grandis* displayed higher chlorophyll amount (TChl, ChlA, ChlB) on days 2 and 4, while these parameters remained stable in *E. dunnii*. On the other hand, the absence of significant chlorophyll loss is an indicator of stress tolerance by these species.

### TSP, proline, and sugars

Regarding potential protective mechanisms, a notable increase in total soluble protein (TSP) content was observed in *E. grandis* on day 4, while it remained relatively stable in *E. dunnii*. This disparity suggests distinct protein synthesis strategies between the two species. The elevated TSP in *E. grandis* may be associated with the synthesis of regulatory and functional proteins, such as Late Embryogenesis-abundant (LEA) proteins, Heat Shock Proteins (HSPs), Cold-Inducible Proteins (KINs), and other cold stress-related proteins (Seki et al., 2002).

Proline, a compatible osmolyte known to accumulate in plant cells, offers protection against dehydration and lipid peroxidation under various abiotic stresses (Verslues and Sharma, 2010). In this study, both species exhibited increased proline amounts over time, consistent with previous reports (Kaplan et al., 2007; Liu et al., 2014; Primo-Capella et al., 2022). However, the peak accumulation occurred earlier in *E. dunnii* (day 2) compared to *E. grandis* (day 4).

Unlike *E. dunnii*, *E. grandis* showed a pronounced increase in proline content on day 4, suggesting its pivotal role in this species’ stress response. Similarly, *E. grandis* displayed higher anthocyanin content on day 4. Despite a report that did not detect significant interspecific differences in anthocyanin content between *E. grandis* and *E. dunnii* (Oberschelp et al., 2020), profiles of these flavonoids are known to vary with clone type within species.

On the second day, *E. dunnii* exhibited significantly higher levels of TSS and phenolics than *E. grandis.* Our findings regarding TSS are consistent with those of Oberschelp et al. (2020) but diverge from the results of Floriani et al. (2013), who did not observe significant differences between the two species. Conversely, our results for phenolics contradict those of Oberschelp et al. (2020), as they reported no significant differences on the concentration of these metabolites between *E. grandis* and *E. dunnii*.

Akin to proline, sugars also act as compatible osmolytes, playing an essential role in protecting cells from chilling injury (Couée et al., 2006; Yuanyuan et al., 2009; Tarkowski and Wim, 2015). In the present work, TSS concentration remained relatively stable among days in both species. In contrast, other authors have observed increased sugar amount after 5 days of cold treatment (Mokochinski et al., 2018; Oberschelp et al., 2022). These results could be explained by shorter cold exposure in the current experiments, variations in stress intensity or different relative importance of defense mechanisms in the clones evaluated.

### Total phenolics and anthocyanins

Plants possess diverse light-absorbing metabolites, phenolic compounds, including anthocyanins, which protect photosynthetic tissues from photooxidative stress caused by excessive light energy. These metabolites function as photochemical energy dissipators, converting excess absorbed light into heat. This mechanism provides an alternative to the xanthophyll cycle, offering an additional layer of photoprotection (Close and Beadle, 2003; Tarkowski and Wim, 2015; Naing et al., 2017; Gould et al., 2018; Kumar et al., 2023).

In the present work, anthocyanin concentration did not change between days of cold treatment in any of the species. Anthocyanins confer a beneficial role when plants are unable to dissipate excess thermal energy and require additional mechanisms to quench excessive light, particularly under prolonged exposure to intense full-spectrum white light or natural sunlight at low temperatures (Gould et al., 2018). Our study did not detect significant changes in chlorophyll or carotenoid amounts in either *E. grandis* or *E. dunnii* across the experimental days. These findings suggest that the existing pigment quantity may be sufficient to prevent photodamage. Alternatively, the relatively low irradiance intensity and sample harvest frequency may not have been sufficient to afford measurable changes in anthocyanin levels.

The concentration of phenolics increased in *E. dunnii*, while levels remained stable in *E. grandis* across the experimental days. The elevated phenolic levels in *E. dunnii* suggest that the accumulation of these compounds may represent a significant mechanism for mitigating photoinhibition in this species. These results differ from those reported by Oberschelp et al. (2020) and Oberschelp et al. (2022), who found an increase in phenolics in both species under cold stress conditions. Again, this may reflect distinct experimental conditions and/or genetic differences in defense strategies among eucalypt commercial clones.

### Comparison of cold-response profiles and phenolic metabolomics

Our results indicate distinct cold tolerance strategies in the clones of *E. grandis* and *E. dunnii*. Notably, the timing of protective mechanism activation differed between the two species. *Eucalyptus dunnii* initiated these responses after a 2-day exposure period, whereas *E. grandis* did so only after 4 days. Furthermore, the specific protective parameters deployed by each species also differed. Data point out to *E. dunnii* primarily relying on the accumulation of sugars and phenolic compounds, with *E. grandis* favoring accumulation of proline and anthocyanins.

Indeed, Principal Component Analysis (PCA) of the biochemical parameter dataset further corroborates these divergent cold tolerance strategies between the two species. In line with this finding, the Partial Least Squares-Discriminant Analysis (PLS-DA) score plot derived from metabolomic analysis revealed a clear species-specific effect across the metabolome. Furthermore, this effect was greater than the differences between cold temperatures.

The phenolic compounds primarily responsible for the observed discrimination were flavonols, ellagitannins, gallotannins, flavones, phenolic acids, and cyclitol phenolics, where flavonols constituted the most abundant class of identified metabolites (Supplementary Figure S1). All these metabolites have been previously reported in *Eucalyptus* leaves (Mokochinski et al., 2018; Noleto-Dias et al., 2023) and wood (Santos et al., 2013). Evidence that phenolic compounds are temperature-responsive metabolites was previously shown by Mokochinski et al. (2018), comparing *E. pellita*, *E. grandis*, and *E. dunnii*. Some polyphenols identified by the authors’ metabolomic analysis increased after low temperature exposure, particularly in the case of *E. dunnii*. In the present study *E. dunnii* 2-day cold treatment showed an accumulation of kaempferol and quercetin glycosides. These flavonols are known to absorb UVB light and play a significant role in photodamage avoidance (Ryan et al., 2002). Flavonols can contribute to mitigate damage caused by cold-induced photoinhibition (Mishra et al., 2023). Indeed, accumulation of flavonols and phenolic acids is a well-established adaptive response to chilling in plants (Rao and Zheng, 2025).

Taken together, overall data highlight that the accumulation of TSS and phenolic compounds is a distinctive cold tolerance strategy of *E. dunnii* and that phenolic fingerprint under cold stress differs in *E. dunnii* and *E. grandis*. Moreover, the latter species seems to rely more intensely on proline and anthocyanin accumulation.

### Considerations on distinct methodology comparisons

The leaf disk-based method herein described is considerably faster and demands less infrastructure for its implementation. Similar approaches to investigate the impact of tree stress on secondary metabolism have been used in previous studies, mostly showing agreement with whole plant data (e.g., De Costa et al., 2013; Palma et al., 2023). A leaf disk method also proved valuable to estimate frost tolerance variability in *Eucalyptus regnans* F. Muell. and *Eucalyptus nitens* (Deane & Maiden) Maiden as a tool for breeding purposes (Raymond et al., 1992). However, like any other experimental method, it is not free of some limitations (Piotrowska and Kacperska, 1990). Among these, one could list the lack of correlative influences among plant organs, the absence of whole plant metabolic coordination, and the non-adequacy to long time span assays. Nonetheless, useful as well as novel information can emerge from the approach used in the present study, particularly for direct comparisons, as described above. Indeed, several consistencies with available literature regarding cold responses in eucalypts were recorded. Admittedly, caution should be always exercised before extrapolating data from simplified to more complex experimental approaches (e.g., organs vs. whole plants, laboratory versus field tests).

## Conclusion

Distinct cold tolerance biochemical strategies were found in *E. grandis* and *E. dunnii* leaf tissues exposed to mild temperate climate winter cold conditions. These differences encompass both the specific protective agents employed by each species and the timing of protective mechanism deployment. Whereas *E. dunnii* appeared to rely predominantly on the accumulation of sugars and phenolic compounds, the accumulation of proline and anthocyanins was primarily present in *E. grandis*. Moreover, 16 phenolic compounds with high discriminant potential were identified, allowing for the differentiation of the cold response in these two species and providing potential future tools for cold tolerance screening. Further studies will delve in detailed exploration of the metabolites and genes taking part in cold tolerance responses in commercial clones of these high interest forest species.

## Data Availability

The raw data supporting the conclusions of this article will be made available by the authors, without undue reservation.
