# Supplementary material for: Cold-induced biochemical changes in leaves of two commercial clones of Eucalyptus
Source: Front Mol Biosci. 2025 Jun 4;12:1584132. doi: 10.3389/fmolb.2025.1584132 (PMC12174460; doi:10.3389/fmolb.2025.1584132)
Supplement: Supplementary file 1 [file Supplementaryfile1.docx]

Supplementary Material

**Supplementary Table S1**. Sensitivity, specificity, and classification error values obtained by PLS-DA 8 LVs.

| **Class** | **Sensitivity (%)** | **Specificity (%)** | **Classification error (%)** |
| --- | --- | --- | --- |
| EG0 | 100 | 100 | 0 |
| EG2 | 100 | 100 | 0 |
| EG4 | 100 | 100 | 0 |
| ED0 | 100 | 100 | 0 |
| ED2 | 100 | 100 | 0 |
| ED4 | 100 | 100 | 0 |

|  | |
| --- | --- |
| R1=OH R2=H R3=OH | Quercetin (flavonol) |
| R1=H R2=H R3=OH | Kaempferol (flavonol) |
| R1=OH R2=OH R3=OH | Myricetin (flavonol) |
| R1=H R2=H R3=H | Apigenin (flavone) |

**Supplementary Figure S1**. Structures of the identified flavonoids. They are present in monoglycosylated forms.
